# A 1-week sleep and light intervention improves mood in premenstrual dysphoric disorder in association with shifting melatonin offset time earlier

**DOI:** 10.1007/s00737-022-01283-z

**Published:** 2022-12-15

**Authors:** Barbara L. Parry, Charles J. Meliska, L. Fernando Martinez, Ana M. Lopez, Diane L. Sorenson, Sharron E. Dawes, Jeffrey A. Elliott, Richard L. Hauger

**Affiliations:** 1 Department of Psychiatry, University of California, 9500 Gilman Dr., La Jolla, La Jolla, CA 92093-0804 USA; 2 Center for Circadian Biology, San Diego, CA USA; 3 Center for Behavior Genetics of Aging, San Diego, CA USA; 4 Center of Excellence for Stress and Mental Health (CESAMH), VA, San Diego Healthcare System, San Diego, CA USA

**Keywords:** Premenstrual dysphoric disorder, Sleep, Wake therapy, Light treatment, Melatonin circadian rhythms

## Abstract

To test the hypothesis that 1 week of combined sleep and light interventions (SALI), which phase-advance (shift earlier) melatonin circadian rhythms, improves mood significantly more than phase-delay (shift later) SALI. After a 2-month diagnostic evaluation for premenstrual dysphoric disorder (PMDD per DSM-5 criteria) in a university clinical research setting, 44 participants enrolled in baseline studies were randomized in the luteal phase at home to (A) a phase-advance intervention (PAI): 1 night of late-night wake therapy (LWT: sleep 9 pm–1 am) followed by 7 days of the morning (AM) bright white light (BWL), or (B) a phase-delay intervention (PDI): 1 night of early-night wake therapy (EWT: sleep 3–7 am) plus 7 days of the evening (PM) BWL. After a month of no intervention, participants underwent the alternate intervention. Outcome measures were mood, the melatonin metabolite, 6-sulfatoxymelatonin (6-SMT), and actigraphy (to assess protocol compliance). At baseline, atypical depression correlated positively with phase delay in 6-SMT offset time (*r* = .456, *p* = .038). PAI advanced 6-SMT offset from baseline more than PDI (*p* < .05), and improved raw mood scores more than PDI (*p* < .05). As hypothesized, percent improvement in mood correlated positively with a phase advance from baseline in 6-SMT offset time (*p* < .001). Treatment with 1 night of advanced/restricted sleep followed by 7 days of AM BWL (PAI) was more efficacious in reducing PMDD depression symptoms than a PDI; mood improvement occurred in association with phase advance in 6-SMT offset time. Combined SALIs offer safe, efficacious, rapid-acting, well-tolerated, non-pharmacological, non-hormonal, affordable, repeatable home interventions for PMDD. Clinical Trials.gov NCT # NCT01799733.

**Supplementary information:**

The online version contains supplementary material available at 10.1007/s00737-022-01283-z.

## Introduction

A potentially disabling condition, premenstrual dysphoric disorder (PMDD) causes extensive personal suffering, occupational impairment, and disruption of interpersonal and family relationships. While 20–80% of women report mood, cognitive, and behavioral disturbances associated with their menstrual cycle (Hamilton et al. 1984; Parry and Wehr 1987), PMDD can progress from premenstrual syndrome (PMS) to major depression (MD) (Halbreich and Endicott 1985; Hamilton et al. 1984), increase risks for peripartum and menopausal depression (Parry et al. 1995), and exacerbate bipolar illness (Dias et al. 2011; Payne 2011). Premenstrual mood symptoms meet criteria for a mental disorder in 5–8% of menstruating women (Accortt et al. 2008; American Psychiatric Association 2013; Yonkers et al. 2008). Current PMDD treatments are only marginally efficacious (< 60% response) (Halbreich et al. 2006), and many women do not want to use, or cannot tolerate, taking chronic antidepressant medications for a periodic illness. Pharmacological interventions are not efficacious in over 40% of women (Halbreich et al. 2006), are associated with problematic side effects, increase the risk of breast and ovarian cancer (Cosgrove et al. 2011), and do not improve health outcomes (Borenstein et al. 2007). Consequently, enhanced PMDD treatments are needed (Borenstein et al. 2007).

A single night of total or partial “wake therapy” (WT) (previously called therapeutic sleep deprivation) produces a rapid, albeit transient, antidepressant response in 40–60% of patients (Giedke and Schwarzler 2002; Schilgen and Tolle 1980; Wirz-Justice and Van den Hoofdakker 1999; Wirz-Justice and Terman 2012; Wirz-Justice et al. 2013; Wu and Bunney 1990). Early-night wake therapy (EWT: i.e., remaining awake until 3:00 am, then sleeping from 3:00–7:00 am), or late-night wake therapy (LWT: i.e., sleeping from 9:00 pm–01:00 am and remaining awake until the following night), benefits mood as much as “total-night” WT (Leibenluft and Wehr 1992; Parry and Wehr 1987; Wirz-Justice et al., 2013). LWT is more efficacious than EWT in some, but not all, MDs (Parry and Wehr 1987; Wirz-Justice et al. 2013). We confirmed wake therapy efficacy in PMDD (Parry and Wehr 1987; Parry 1995) and peripartum depression (Parry et al. 2000, 2019).

Light treatment sustains wake therapy benefits often lost after recovery sleep, and wake therapy hastens and potentiates light treatment effects taking 5–10 weeks to significantly improve mood in peripartum non-seasonal depression (Corral et al. 2000, 2007; Epperson et al. 2004; Oren et al. 2002; Wirz-Justice et al. 2011). We previously reported and replicated the independent antidepressant effects of light (Parry et al. 1989; 1993; 1997a) and wake therapy (Parry and Wehr, 1987; Parry et al. 1995) in PMDD. AM light significantly improved scores on the Hamilton Rating Scale for Depression (HRSD) by 50% (Parry et al. 1997a), replicated using a shorter, brighter light pulse (Parry et al. 2011). LWT significantly reduced mean HRSD depression scores by 62.2% (Parry et al. 1995). In the current study, we combined wake and light interventions to enhance their individual benefits.

As misaligned circadian rhythms characterize mood disorders (Goel et al. 2013; Monteleone et al. 2011; Srinivasan et al. 2006; Wehr and Wirz-Justice 1982), we sought to test the hypothesis that wake and light interventions exert antidepressant effects by correcting misaligned circadian rhythms (CRs), best measured by melatonin in humans. In PMDD, we previously found plasma melatonin CR were phase delayed (shifted later) in the symptomatic luteal, compared with the asymptomatic follicular, menstrual cycle phase, which correlated with more depressed mood (Parry et al. 1997b, 2008a). Consistent with the internal coincidence model of sleep deprivation and depression (Wehr and Wirz-Justice 1981), baseline timing disturbances between sleep and melatonin became normalized after treatment (Parry et al. 1989, 1997a, 1999). We reasoned that advancing and restricting sleep with 1 night of LWT followed by 7 days of the morning (AM) bright white light (BWL), which phase advances CRs (active intervention), would benefit mood more than delaying and restricting sleep with a single night of EWT followed by 7 days of the evening (PM) BWL, which phase delays CRs (control intervention).

## Methods

### Overview

**Fig. 1:**
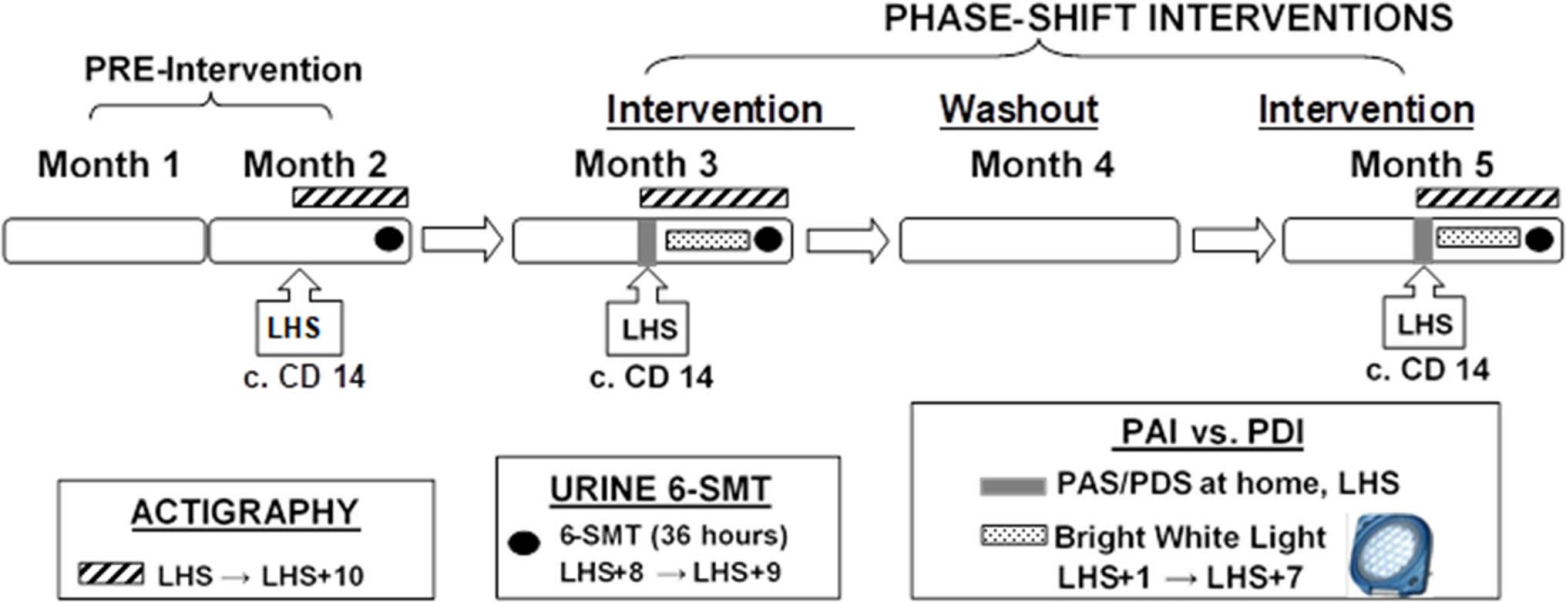
Study flow: Luteinizing hormone surge (LHS) marks ovulation. Women begin actigraphy recordings on LHS for 10 days on menstrual cycle days (CD) 14–24. In months 2, 3, and 5, beginning on CD 14, women collect urine samples for 6-sulphatoxymelatonin (6-SMT) for 36 h over 2 nights between 8 and 9 days after the LHS (c. CD 22–23). They undergo phase-advance sleep (PAS) or phase-delay sleep (PDS) at home on the night of the LHS, and receive bright morning (AM) or bright evening (PM) light on days 1–7 after LHS (CD 15–21) in a counterbalanced, cross-over design in months 3 and 5. Mood ratings (Structured Interview Guide for the Hamilton Rating Scale for Depression (HRSD) with Atypical Depression supplement-SIGH-ADS) are obtained as follows: months 1 and 2—once per week; months 3 and 5—LHS (Pre-PAI/PDI), daily LHS + 1 day → LHS + 2 days, and once during the evening after last treatment day (LHS + 7/8 days). Blind raters assessed mood by the SIGH-ADS once during month 4 and once a month after intervention 2 for 3 months to clinically monitor relapse

We described the essential features of our protocol including screening, eligibility, inclusion, and exclusion criteria previously (Parry et al. 1995, 1997a,b, 2008a). In the present study, we used a randomized-order, crossover design, studying NC and PMDD women during luteal phases of two separate menstrual cycles. Using a “crossover” design, we tested each participant twice, with two contrasting interventions separated by 1 month. For the first intervention, half the subjects were assigned to a phase advance intervention (PAI): (1) A partial night (4 h) of phase-advancing sleep (sleep 9 pm–1 am, followed by wakefulness), followed by 7 consecutive mornings of AM BWL (light-emitting diode-LED administered for 60 min) within 30 min after wake time; or (2) a phase delay intervention (PDI): A partial night (4 h) of phase-delaying sleep (remain awake until 3 am, then sleep 3–7 am), followed by 7 consecutive evenings of PM BWL (administered for 60 min), starting 90 min before anticipated sleep onset. See Fig. 1. We assessed intervention effects on (1) the urinary melatonin metabolite, 6-sulfatoxymelatonin (6-SMT); (2) mood; and (3) sleep/activity (actigraphy, to confirm protocol compliance). We tested the following hypotheses (H):

1. PAI vs. PDI will normalize melatonin circadian rhythms in PMDD by phase-advancing urinary melatonin timing measures (i.e., 6-SMT offset time).
2. PAI will improve mood more than PDI.
3. Mood improvement will correlate positively with the magnitude of phase-advance in 6-SMT offset time after PAI.

### *Statistical analyses*

For inferential tests of H1 and H2, we used a 2 × 2, within subjects/repeated measures Analyses of Variance to compare the effects of PAI vs. PDI on PMDD mood, 6-SMT timing, and sleep measures, before and after interventions. For H3, we evaluated the relationships of changes in mood and sleep to changes in 6-SMT timing using Pearson correlations.

### Procedures

We received 688 calls from women interested in participating. We screened 368 via telephone and scheduled 137 for screening visits. A total of 85 women enrolled in the screening phase and 44 (29 PMDD + 15 NC) were randomized to intervention from 5/1/13 to 4/30/18. Of these, 15 PMDD and 8 NC completed the entire protocol and two participants completed at least one intervention arm.

The University of California San Diego Institutional Review Board approved the protocol, and all participants gave written informed consent after procedures were explained fully. Participants were without alcohol abuse, significant medical illness, or medication that would interfere with study measures.

To establish DSM-IV-TR (APA, 2000) entrance and baseline criteria, trained clinicians used the Structured Clinical Interview for DSM-IV (SCID) (First et al.^1995^) and assessed pre- and post-treatment mood (see Fig. 1 legend) with the Structured Interview Guide for the Hamilton Rating Scale for Depression (HRSD) with Atypical Depression supplement (SIGH-ADS) (Williams and Terman 2003); participants completed 2 months of daily mood ratings in which**,** for study inclusion, PMDD participants were required to be asymptomatic during the follicular menstrual cycle phase, and demonstrate a 30% increase in symptoms in the luteal phase. From the pool of volunteers, we obtained mood data on PMDD and essentially asymptomatic, normal control (NC) women. We excluded patients with substance use, bipolar, and primary anxiety disorders.

#### Documenting sleep time

We documented compliance with sleep and wake protocol requirements by actigraphy and by requiring participants to telephone the laboratory every 30 min while awake between 9 pm and 7 am.

#### Light Box

Subjects sat before a portable (5.5″ × 6.25″) Litebook® light box (an array of 60 cool white light-emitting diodes behind a clear plastic screen with an intensity of 1350 lx and an irradiance of 2.41 × 10^−9^ w/cm^2^ at a distance of 21 in, and spectral emission peaks at 464 nm and 564 nm) (The Litebook Company Ltd., Alberta, Canada) for 60 min. Participants did not stare directly at the light source as it could cause discomfort, but is not harmful. The distance of the subject from the light source was calibrated individually for each light box using a Meterman LM631 Digital Light Meter (Meterman Test Tools, Everett, WA) to ensure an intensity of 1350 lx at 21 in. We provided a measuring tape to ensure the proper distance between the light source and the subject. Ambient light intensity and spectra were documented by the Actiwatch Spectrum.

#### Dependent Measures

For a complete description of 6-SMT and actigraphy measures, please see Supplementary Online Resource for Archives of Women’s Mental Health.

## Results

Baseline demographic characteristics of study participants are reported in Table 1.

**Table 1:** Baseline demographic characteristics of study participants. Ethnicity: normal control (NC) = Asian (1), Hispanic (2), Caucasian (2); premenstrual dysphoric disorder (PMDD) = Asian (4), Hispanic (4), Caucasian (7)

	Normal control (*N* = 5)			PMDD (*N* = 15)		Range	*P*
	Mean	S.D	Range	Mean	S.D		
Age (years)	32.7	± 4.1	24–37	34.9	± 6.2	18–44	ns
6-SMT acrophase^3^	3.67	± 1.03	1.8–4.7	3.40	± 2.02	1.3–4.9	ns
6-SMT onset^3^	22.1	± 1.4	21.0–24.5	22.6	± 1.7	21.2–24.4	ns
6-SMT offset^3^	8.1	± 2.0	7.1–10.9	8.2	± 1.9	5.8–11.5	ns
Sleep onset time^3^	23.6	± 1.4	23.2–24.7	23.9	± 1.5	21.0–25.5	ns
Sleep end time^3^	7.7	± 0.6	7.3–8.6	7.6	± 1.6	7.3–8.6	ns
Total sleep time (min)	436.4	± 48.5	412–460	424.1	± 59.6	282–509	ns
Baseline BDI^1^ score	0.6	± 0.8	0.0–1.5	5.0	± 6.3	0.0–9.0	.046
Baseline SIGH-ADS^2^ score	4.7	± 2.0	4.5–8.5	23.6	± 8.4	11.5–35.3	.001

^1^Beck Depression Inventory (BDI)

^2^Structured Interview Guide for the Hamilton Depression Rating Scale with Atypical Depression Supplement (SIGH-ADS)

^3^Decimal Hours (h) to indicate the unit of measurement

### Protocol deviation

Some PMDD participants (9 of 24) deviated from the design protocol by not self-administering morning light during the active PAI (LWT + AM BWL) portion of the study. A comparison of improvements (percent changes) from baseline mood measures in those who fully complied (*N* = 15) vs. those who did not (*N* = 9) showed women who self-administered morning light per protocol experienced significantly greater improvements (percent reductions from baseline) in HRSD (71.5 vs. 42.5%, *p* = 0.001), atypical (83.0 vs. 48.0%, *p* = 0.003), and SIGH-ADS (76.2 vs. 48.0%, *p* = 0.002) scores. Thus, as expected, adding bright white light exposure for 1 week after wake therapy increased mood benefits significantly beyond those achieved with wake therapy alone. (The remainder of this report focuses entirely on outcomes of the 15 women who complied with both wake + light interventions.)

**Fig. 2:**
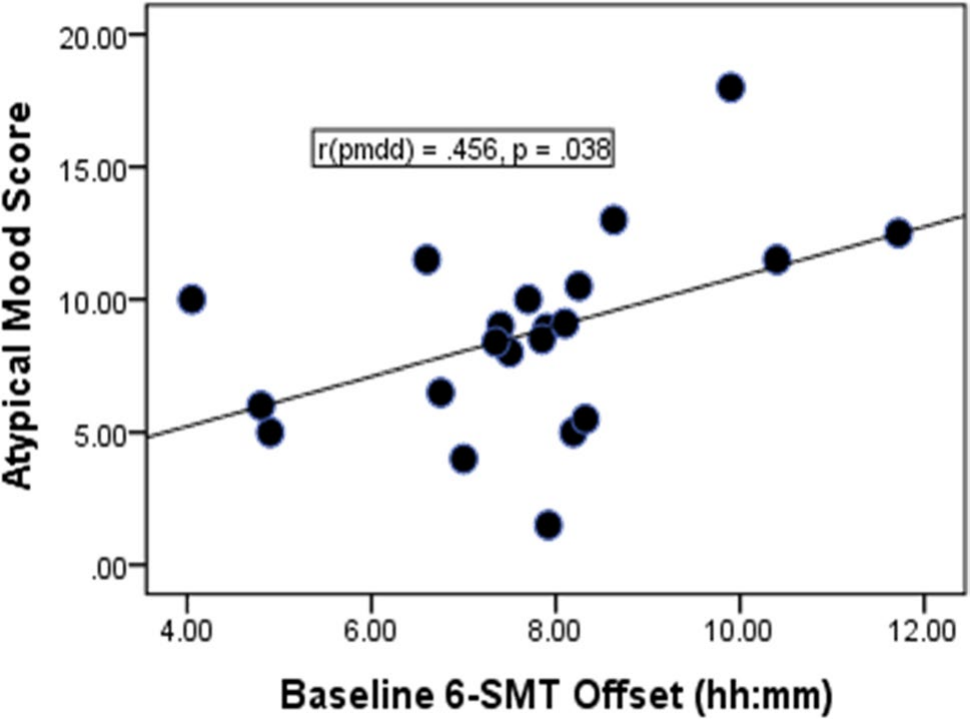
At baseline, greater depressed atypical mood score of the Structured Interview Guide for the Hamilton Rating Scale for Depression (HRSD) with Atypical Depression supplement (SIGH-ADS) score correlated positively with greater phase-delay in 6-sulfatoxymelatonin (6-SMT) offset (*r* =  + .456, *p* = .038)

At baseline, the cosine-derived 6-SMT offset was significantly correlated with the atypical mood score of the SIGH-ADS in PMDD + NC combined (*r* =  + 0.456, *p* = 0.038; see Fig. 2); i.e., greater phase-delay in 6-SMT offset was associated with greater atypical depressed mood (e.g., symptoms of increased appetite, weight gain, sleepiness, and fatigue).

### PAI vs. PDI effects on 6-SMT timing

Post-intervention, MANOVA showed the PAI advanced 6-SMT timing from baseline more than PDI (*F*(3,18) = 4.04, *p* = 0.023 for trend). Simple effects analysis showed, as hypothesized (H1), advance in 6-SMT offset was significantly greater after PAI vs. PDI (mean + / − SD =  + 0.84 + / − 1.36 vs. + 0.28 + / − 2.35 h, *p* < 0.001). Post-intervention changes in 6-SMT onset and acrophase did not differ significantly (*p* > 0.05).

#### Mood Effects of PAI vs. PD

**Fig. 3:**
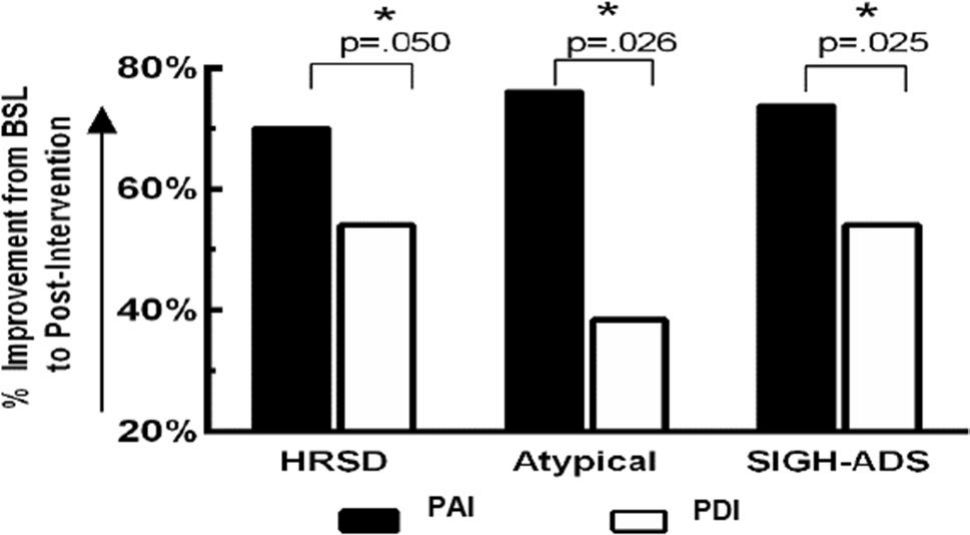
Percent change (improvement from baseline) in mood measures was significantly greater after phase-advance intervention (PAI) vs. phase-delay intervention (PDI) for Hamilton Rating Scale for Depression (HRSD), Atypical, and Structured Interview Guide for the Hamilton Rating Scale for Depression (HRSD) with Atypical Depression supplement (SIGH-ADS) scores (all *p* < .05)

Both PAI and PDI improved objective, interview-assessed depression; however, as hypothesized, PAI improved mood significantly more than PDI on HRSD, atypical, and SIGH-ADS indices (see Fig. 3.)

### Relationship of mood change to 6-SMT change

**Fig. 4:**
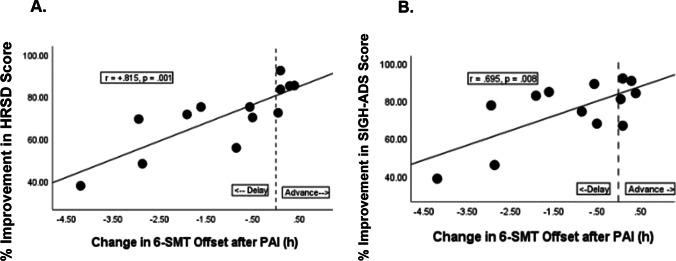
Advance in 6-sulfatoxymelatonin (6-SMT) offset time after phase-advance intervention (PAI) correlated positively with magnitude of improvement in **A** Hamilton Rating Scale for Depression (HRSD) (*r* =  + .815, *p* = .001) and **B** the Structured Interview Guide for the Hamilton Rating Scale for Depression (HRSD) with atypical depression supplement (SIGH-ADS) score (*r* =  + 0.695, *p* = .008)

The mean percent change from baseline was significantly greater after PAI (the active condition) vs. PDI (control condition) for atypical mood (88.95 + / − 17.1 vs. 58.49 + / − 43.6%, *p* = 0.028%) and SIGH-ADS score (75.77 + / − 17.8 vs. 63.61 + / − 21%, *p* = 0.025). Furthermore, as hypothesized (H3), advance in 6-SMT offset time after PAI was positively correlated with the magnitude of improvement in HRSD score (*r* =  + 0.815, *p* = 0.001) and SIGH-ADS score (*r* =  + 0.695, *p* = 0.008). Thus, greater mood improvement after PAI was associated with greater phase advance in 6-SMT offset time (see Fig. 4A, B). In contrast, after the PDI, the change in 6-SMT offset did not correlate significantly with improvement in HRSD, atypical, or SIGH-ADS score (all *p* > 0.05).

#### Sleep effects of PAI vs. PDI.

Neither PAI nor PDI produced significant changes from baseline in sleep parameters (timing of sleep onset, offset, mid-sleep, or total sleep time); nor were any sleep changes significantly correlated with mood or 6-SMT changes.

## Discussion

The aim of this study was to determine whether PMDD depressed mood could be improved using phase-advancing sleep and light intervention (SALI) to correct the phase delay in melatonin circadian rhythms (CR) we found in PMDD (Parry et al. 1997a, 2008a). We hypothesized that since estradiol advances, and progesterone delays CR (Albers et al. 1981; Morin et al. 1977), fluctuations in these hormones during the menstrual cycle could dysregulate CR, and correcting CR misalignment could thereby improve depressed mood. In support of this hypothesis, we found the following: (1) In the baseline luteal phase, PMDD atypical depression symptoms were associated with a greater phase delay of 6-SMT offset; (2) A phase-advance intervention (PAI: late-night wake therapy-LWT plus AM bright white light-BWL) produced significantly greater mood improvement than a phase-delay intervention (PDI: early-wake therapy-EWT plus PM BWL); and (3) After PAI, improvement in HRSD and SIGH-ADS mood scores correlated significantly with phase advance in 6-SMT offset.

Importantly, we designed the study arms to create identical sleep restriction durations (4 h), plus identical bright light exposures (60 min/day for 7 days), but at different times of day, to effect maximal CR realignment. Thus, the treatments varied only in the times of day when sleep restriction and light exposure were instituted, with PAI designed to advance 6-SMT timing vs. PDI. That mood benefits and 6-SMT phase-advanced timing were, indeed, significantly correlated in the PAI vs. PDI study arms confirmed that hypothesis.

We discuss implications of these findings in relation to previous literature below.

1. Phase delay in CRs characterizes other depressive disorders. Lewy et al. (2006) reported phase-delayed melatonin CR in seasonal affective disorder; we found phase-delayed plasma melatonin CR in postpartum and menopausal depressed participants (Parry et al. 2008b, c); and Tuunainen et al. (2002) found phase-delayed 6-SMT rhythms in post-menopausal depression. Phase-delayed CR, or delayed chronotype, also were associated with later sleep onset times in peri- and post-menopausal women (Meliska et al. 2011). One possible explanation is that later sleep onset times and chronotypes reflect later wake times, which prevents CR synchronization from morning bright light. The resulting misalignment of CR then contributes to depressive mood**,** according to the internal coincidence model of sleep and depression (Wehr and Wirz-Justice 1981). Under the influence of progesterone in the luteal phase, both PMDD and normal control women delay melatonin CR in the luteal vs. follicular menstrual cycle phase; but only PMDD women experience mood symptoms then (Parry et al. 2008a), suggesting that individuals with depressive histories are more vulnerable to developing symptoms during acute phase shifts, as occurs in jet lag (Parry 2002). That symptoms remit in the early follicular phase when phase-advancing estradiol increases and phase-delaying progesterone decreases (as with sleep and light interventions that restore a CR phase advance in the luteal phase), suggests CR abnormalities in PMDD are more state, than trait, dependent. Also, noteworthy is that baseline phase-delayed CR were associated with atypical symptoms that characterize depressions associated with the reproductive cycle and seasonal affective disorder (Parry et al. 1987). These symptoms are particularly responsive to light therapy (Terman et al. 1989).
2. That the PAI, a phase-advancing SALI, reduced depression scores significantly more than the PDI, a phase-delaying SALI, confirms the hypothesis that phase-advancing CR is particularly effective in reducing PMDD depressed mood. Mood improvement after light treatment has been associated with a corrective phase shift of melatonin CRs in other depressive disorders: by Epperson et al. (2004) with bright light therapy for antepartum depression; by Terman et al. (2001) in winter depression; with the activity rhythm in unipolar depression (Dallaspezia et al. 2012); and in other mood disorders (Benedetti et al. 2005; Bloching et al. 2000; Colombo et al. 2000; Even et al. 2008; Fritzsche et al. 2001; Golden et al. 2005; Kripke et al. 1983; Loving et al. 2002; Neumeister et al. 1996; Parry and Maurer 2003; Riemann et al. 1999; Sokolski et al. 1995; Tuunainen et al. 2004; van den Burg et al. 1990; Wehr et al. 1985; Wu et al. 2009), thereby potentially providing new approaches to investigate and treat PMDD.
3. The correlation of mood improvement magnitude with 6-SMT phase advance after PAI suggests the intervention engages a relevant pathogenesis: phase-delayed melatonin in the luteal menstrual cycle phase.

These results suggest potential benefits for other reproductively related mood disorders when a circadian pathophysiology is identified. For example, in pregnancy, we showed plasma and urinary melatonin were phase advanced in depressed vs. healthy participants (Parry et al. 2008b), and mood improved significantly more after phase-delaying than phase-advancing SALI. In contrast, in postpartum-depressed participants plasma and urinary melatonin were phase delayed (Parry et al. 2008b), and mood improved more after phase-advancing than phase-delaying SALI. In menopausal, like postpartum-depressed participants, plasma and urinary melatonin were phase delayed (Parry et al. 2008c), and mood improved more after phase-advancing vs. phase-delaying SALI. Thus, each reproductively related depressive disorder has a specific chronobiological abnormality that provides a basis for optimizing interventions targeted to specific pathogenic mechanisms.

The advantage of combining sleep and light interventions is that the sleep intervention hastens and potentiates mood benefits of light treatment, and light treatment maintains mood benefits of sleep intervention, which otherwise may be lost after a night of recovery sleep (Wirz-Justice 2011). Initially, we used the sleep intervention alone in PMDD (Parry and Wehr 1987; Parry et al. 1995) where, in contrast to other mood disorder**s**, women with PMDD did not relapse after a night of recovery sleep**;** i.e., they were more night 2, rather than night 1, responders, which may indicate a more serotonergic, rather than noradrenergic, pathophysiology, supported by extensive studies (see review Parry 2001). As light treatment alone in non-seasonal depression may require at least 5 weeks to improve mood significantly (Parry and Maurer 2003), light treatment, without the priming effect of wake therapy, is less efficacious for PMDD when administered during the necessarily limited time period of 7 days in the symptomatic late luteal phase (Parry et al. 1989, 1993). The advantage of combining interventions is that sleep interventions may produce rapid improvements, which are subsequently sustained by light interventions.

These sleep and light interventions are safe, efficacious, rapid-acting, affordable, readily repeatable, well-tolerated with few side effects, non-pharmacological and non-hormonal home treatments that can be administered by paraprofessionals without office or clinic visits.

Study limitations include a relatively small sample size, from only a single study site, and melatonin measured by 6-SMT rather than plasma. Further, as 37.5% of participants did not complete the full protocol, we confined conclusions about primary study outcomes to results of only participants who completed the full protocol. Although in our earlier study of wake therapy (Parry et al. 1995), the duration of benefit lasted up to 6 months, the durability of sleep + light benefits in the present study needs to be tested.

## Supplementary information

Below is the link to the electronic supplementary material.

Supplementary file1 (DOCX 27 KB)
