# Supplementary material for: A 1-week sleep and light intervention improves mood in premenstrual dysphoric disorder in association with shifting melatonin offset time earlier
Source: Arch Womens Ment Health. 2022 Dec 15;26(1):29–37. doi: 10.1007/s00737-022-01283-z (PMC9908689; doi:10.1007/s00737-022-01283-z)
Supplement: Supplementary file 1 — Supplementary file1 (DOCX 27 KB) [file 737_2022_1283_MOESM1_ESM.docx]

**Supplementary Online Resource for Archives of Women’s Mental Health**

A One-Week Sleep and Light Intervention Improves Mood in Premenstrual Dysphoric Disorder in Association with Shifting Melatonin Offset Time Earlier

Barbara L. Parry MD, Charles J. Meliska PhD, L. Fernando Martinez BA, Ana M. Lopez BS, Diane L. Sorenson MPH, Sharron E. Dawes PhD, Jeffrey A. Elliott PhD, Richard L. Hauger MD

From the Department of Psychiatry, University of California, San Diego

Center for Circadian Biology (Drs. Parry, Meliska, Elliott)

Center for Behavior Genetics of Aging (Dr. Hauger)

Center of Excellence for Stress and Mental Health (CESAMH), VA San Diego Healthcare System (Dr. Hauger)

This supplementary material has been provided by the authors to give readers additional information about their work.

**Dependent Measures**

6-sulphatoxymelatonin (6-SMT): Sample Collection

Urinary 6-SMT Assay Methodology

Actigraphy

**6-sulphatoxymelatonin (6SMT): Sample Collection:** A principal melatonin metabolite abundant in urine, urinary 6-SMT is highly correlated with plasma melatonin and readily quantified with an enzyme immunoassay (EIA), thereby serving as an excellent marker for phase response (Kripke et al. 2007; Youngstedt et al. 2019). For urine collections, participants measured and recorded the time and total volume of each urine voiding over a 36-hour interval starting at 6pm one evening and ending at noon the next day, then restarting at 6pm that same day and ending at noon the following day. Two 2 mL aliquots from each voiding were frozen at home in duplicate, using labeled and numbered vials, and later stored at -70 C in the lab until assay. Subjects received thorough instruction regarding accurate recording of time and volume and proper collection and storage of the aliquots associated with each sample, including hands-on experience. Subjects also were encouraged to drink extra fluids during waking hours (~ 200 mL every 2 hours) to remain well-hydrated and to facilitate more frequent voiding. Subjects received an oversupply of duplicate sample vials, each with easily read numbers on the printed label which are repeated on the vial tops to ensure correct identification of each sample with the associated time and volume on the record sheet. They also were supplied with 1000 mL plastic bottles for refrigerator storage of samples collected during awakenings at night (so that volume measurement and transfer to freezer vials could be postponed until morning). The above urine collection methods have served us well for many years (Cole et al. 2002; Kripke et al. 2007; Tuunainen et al. 2002). Since light suppresses melatonin acutely (within 30 minutes), melatonin measures were obtained after completion of the light intervention to avoid confounding the suppressive effects with the phase-shifting effects of light. Based on previous work (Parry et al. 1990, 1997a,b), melatonin profiles are stable and consistent within an individual when measured at different time points. Measuring 6-SMT in urine is less expensive and burdensome to patients compared with plasma measures.

**Urinary 6-SMT Assay Methodology:** Circadian rhythms of 6-SMT excretion were measured using Bühlmann 96 well ELISA kits (EK-M6S) purchased from ALPCO, Diagnostics, Ltd. (Windham, NH) as described previously. (Kripke et al. 2007; Tuunainen et al. 2002) At the usual dilution of 1:200, the analytical sensitivity of this EIA is 0.35 ng/mL and the functional least detectable dose (for coefficient of variation (CV) < 20%) is 1.3 ng/mL. Whenever possible all samples from an individual overnight collection are run at the same time on the same 96-well plate. Samples are assayed in duplicate or re-assayed at either increased or decreased dilution (1:25 to 1: 3200) to obtain more accurate estimates, or to clarify irregular circadian patterns in excretion rate (ng/hr) and outlying values. An advantage of urinary assays is that a much smaller number of assays are needed per 24 h because we compute accurate excretion rates (6-SMT ng/h) from the volume and time lapse associated with each voiding.

**Actigraphy:** We obtained simultaneous measures of physical activity and ambient illumination using the Actiwatch Spectrum ® device for 10 days in the luteal phase starting on the day of the LH surge, during the 2^nd^ month of screening evaluation and during each subsequent probe month. The Actiwatch Spectrum, developed by Respironics®, a subsidiary of Royal Philips Electronics, is a small device (48.5 x 36.7 x 13.8 mm; weight 29.8 g with band) containing a piezoelectric linear accelerometer (sensitive to 0.003 g and above), log-linear photometric transducer (sensitive from <0.01 lux to >100,000 lux), 3 photon flux and irradiance color sensors in the red, green, and blue bands of visible light, and a microprocessor with 2 Mbits RAM memory and associated circuitry that allows for 36 days continuous recording of 1-minute epochs. The orientation and sensitivity of the accelerometer are optimized for highly effective sleep-wake inference from wrist activity. The illumination measurements are roughly log-linear from a range below moonlight to the brightest summer day at noon. An important innovative feature of this device is an off-the-wrist sensor, which distinguishes when the participant has removed the device for any reason. The Actiwatch Spectrum also allows us to determine spectral composition of light, which may differentially affect circadian entrainment including phase-shifts, and therefore, intervention responses (Parry & Maurer 2003).
